# Graphene‐Based Opto‐Electronic Platform for Ultra‐Sensitive Biomarker Detection at Zeptomolar Concentrations

**DOI:** 10.1002/smtd.202402026

**Published:** 2025-01-21

**Authors:** Matteo Piscitelli, Cinzia Di Franco, Giuseppe Valerio Bianco, Giovanni Bruno, Eleonora Macchia, Luisa Torsi, Gaetano Scamarcio

**Affiliations:** ^1^ Dipartimento Interateneo di Fisica Università degli Studi di Bari Aldo Moro 70125 Bari, Italy and CNR IFN Bari 70125 Italy; ^2^ Consiglio Nazionale delle Ricerche – Consiglio Nazionale delle Ricerche – Istituto di Fotonica e Nanotecnologie CNR‐IFN Bari 70125 Italy; ^3^ Consiglio Nazionale delle Ricerche –Istituto di Nanotecnologia CNR‐Nanotech Bari 70125 Italy; ^4^ Dipartimento di Farmacia‐Scienze del Farmaco Università degli Studi di Bari Aldo Moro Bari 70125 Italy; ^5^ Dipartimento di Chimica Università degli Studi di Bari Aldo Moro Bari 70125 Italy; ^6^ NEST Istituto Nanoscienze – CNR and Scuola Normale Superiore Pisa I‐56127 Italy

**Keywords:** biosensors, graphene, Raman spectroscopy, single‐molecule

## Abstract

A ground‐breaking graphene‐based biosensor designed for label‐free detection of immunoglobulin M (IgM) achieving a remarkable concentration of 100 zeptomolar (10^−19^ m), is reported. The sensor is a two‐terminal device and incorporates a millimeter‐wide gold interface, bio‐functionalized with ≈10^12^ anti‐IgM antibodies and capacitively coupled to a bare graphene electrode through a water‐soaked paper strip. In this configuration, few affinity binding events trigger a collective electrostatic reorganization of the protein layer, leading to an extended surface potential (SP) shift of the biofunctionalized Au surface. The SP shift, mediated by electrolyte capacitive coupling, induces a corresponding shift in the Fermi level of graphene. This shifts the graphene phonon frequencies, which are measured by Raman spectroscopy. Decoupling the sensing interface from the transducing graphene layer provides flexibility in surface chemistry modifications, while preserving the graphene integrity. A key aspect of this biosensor is its ability to precisely determine the graphene charge neutrality point from the voltage dependence of phonon frequency shifts, enabling detections of biomarker at unprecedented low concentrations. The integration of graphene with optical probing demonstrates a proof‐of‐concept and establishes a ground‐breaking approach to in situ biomarker detection, setting the stage for a future generation of portable opto‐electronic high‐performance diagnostic tools for single‐marker detection.

## Graphical abstract

An opto‐electronic graphene‐based biosensor detects immunoglobulin M (IgM) at 100 zeptomolar (10^−19^ M) concentration. It features a biofunctionalized gold electrode capacitively coupled to a graphene electrode via a water‐soaked paper strip. Surface potential changes induced by few affinity events are transduced as shifts of the graphene phonon frequencies and detected by Raman spectroscopy.

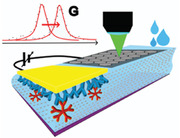

## 1 Introduction

The increasing demand for precise and efficient diagnostic tools in healthcare has spurred interest in developing sensitive and reliable bioanalytical platforms for biomarker detection.^[^ ^1^, ^2^ ^]^ Researchers are actively exploring novel materials and related technologies that enhance sensitivity, specificity, and reliability. Devices based on graphene and other 2D materials have emerged as promising candidates for their ability to detect key‐relevant biomarkers efficiently and selectively, including small molecules,^[^ ^3^, ^4^ ^]^ nucleic acids,^[^ ^5^, ^6^ ^]^ and proteins.^[^ ^7^, ^8^ ^]^ Graphene structural and electronic characteristics, such as a high surface‐to‐volume ratio and superior electrical conductivity, are particularly advantageous for developing label‐free, real‐time biosensing devices that deliver enhanced analytical performance.^[^ ^9^ ^]^ Moreover, graphene is sensitive to chemical and electrostatic changes at its surface, transducing these variations into strong electrical or optical signals, which are crucial for accurate biomarker detection.^[^ ^10^ ^]^

Graphene‐based field‐effect transistors (FETs) are highly sensitive biosensors that exploit the unique properties of graphene. Biomarkers binding to the graphene surface induce a change in the charge carrier density, varying the electrical conductivity. This change can be measured as a shift in the transistor figures of merit, providing quantitative data about the biomarker concentration.^[^ ^11^ ^]^

Electrochemical sensors employ graphene as an electrode material, leveraging its high conductivity and large surface area. Biomarker interactions at the electrode surface result in measurable changes in current, potential, or impedance, making these sensors particularly useful for detecting enzymes, DNA, or redox‐active small molecules.^[^ ^12^ ^]^

Furthermore, graphene's ability to quench fluorescence can be exploited in optical biosensors. When a fluorescently labeled biomarker binds to graphene, its fluorescence is quenched, decreasing the signal in proportion to the biomarker concentration. Alternatively, changes in the light absorption or Raman scattering of graphene upon biomarker binding can also be used for detection.^[^ ^13^, ^14^, ^15^ ^]^

The functionalization protocol is a key process in high‐performance biosensors. It involves biofunctionalizing either the graphene surface or a metallic electrode that is capacitively coupled with graphene. Anchoring bioreceptor molecules, such as DNA, proteins, or enzymes, onto graphene surface is a common practice in graphene‐based electrolyte‐gated FET sensors and allows to reach a concentration on the order of femtomolar (10^−15^ m).^[^ ^16^, ^17^ ^]^ The main disadvantages of biofunctionalized‐graphene‐FET biosensors, include stability and reproducibility issues, as the immobilized biomolecules may degrade or change over time, affecting the sensor reliability.^[^ ^4^, ^16^ ^]^ On the other hand, functionalizing the gate electrode surface has the advantage of decoupling the sensing interface from the transducing graphene layer, providing flexibility in surface chemistry modifications, while preserving the graphene integrity.^[^ ^18^ ^]^ Interestingly, from the sensing‐induced shift of the graphene charge neutrality point (CNP), as extracted by transistor‐like characteristics, a lowest detected concentration of 3.5 × 10^−19^ m is reported.^[^ ^19^ ^]^

In this work, we demonstrate a groundbreaking graphene‐based opto‐electronic biosensor capable of label‐free detection down to the few‐molecule regime. The sensor structure is a two‐terminal device, comprising a millimeter‐wide gold (Au) interface bio‐functionalized with capturing antibodies, capacitively coupled, through a water‐soaked paper strip, to a bare graphene electrode, without the source and drain electrodes needed for transistors. The electrolyte‐soaked paper strip allows stable liquid environment, driven by capillary forces for pump‐free microfluidic platform. The affinity binding events trigger extended surface potential variations on a capturing surface that hosts trillions of recognition sites.^[^ ^20^, ^21^ ^]^ Experimental evidence supporting this amplification mechanism was obtained by kelvin probe force microscopy (KPFM).These electrostatic changes are capacitively transferred from the biolayer to the graphene layer, where they vary the graphene carrier concentration, or equivalently, the graphene Fermi level *E_F_* and the CNP. At variance with previous reports based on the charge transport analysis, we exploit here the strong dependence of graphene phonon energies from the carrier concentration. The *E_F_* shift induce measurable changes Δω_*G*_ of the graphene phonon frequencies^[^ ^22^, ^23^ ^]^ which we readily measure by Raman spectroscopy. A simple model allows to extract the graphene charge CNP from the voltage dependence of Δω_*G*_. Our immunoassay is qualitative showing an OFF/ON type response, confirming that our method is a powerful novel optical approach to detect biomarkers with unprecedented sensitivity.

## 2 Results and Discussion

**Figure 1:**
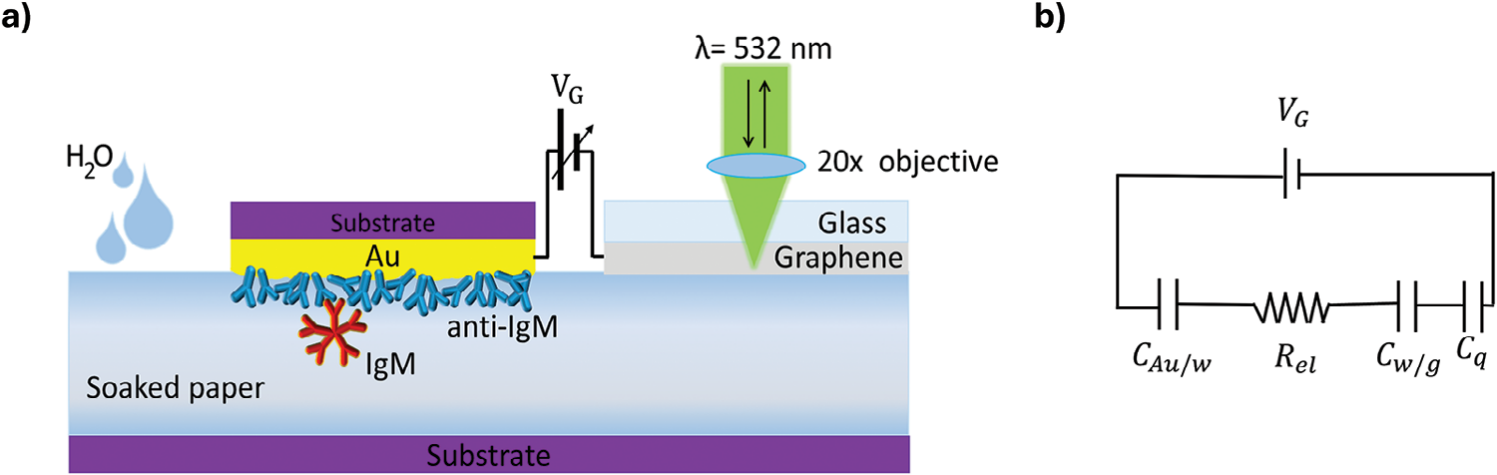
a) Schematic cross‐sectional view of the sensing device, which includes an Au electrode biofunctionalized with an anti‐IgM layer comprising 10^12^ molecules cm^−2^, and a graphene electrode capacitively coupled via a water‐soaked paper strip. An external DC voltage (V_G_) is applied between the Au and the graphene electrodes by a voltage generator. b) Equivalent circuit of the device, including the capacitances of the EDLs at the Au/water (C_Au/w_) and water/graphene (C_w/g_) interfaces, the quantum capacitance of graphene (C_q_) and the resistance of the water strip (R_el_). The Raman spectrum of graphene is recorded by a micro‐Raman spectrometer in a backscattering configuration, using a 532 nm laser.

**Figure** **1**a illustrates the device structure. The sensing element is a thin Au electrode (50 nm‐thick, ≈20 mm^2^ wide), biofunctionalized with a dense physisorbed layer of ≈10^12^ capturing anti‐immunoglobulin M (anti‐IgM) antibodies.^[^ ^24^, ^25^ ^]^ The transducing element is a single layer graphene electrode, grown by chemical vapor deposition and transferred on a transparent glass substrate, which enables optical probing of graphene Raman spectrum. The two electrodes are capacitively coupled through a deionized water layer, sustained by a paper strip, acting as electrolyte as shown in Figure S1 and described in Section S1 (Supporting Information). A DC voltage, V_G,_ is applied to the Au electrode, while keeping the graphene layer grounded. Two electrical double layers (EDLs) form at the Au/water and water/graphene interfaces. In the equivalent circuit depicted in Figure 1b, they are represented as two capacitors with capacitances C_Au/w_ and C_w/g_, respectively. The characteristic quantum capacitance of graphene (C_q_)^[^ ^26^ ^]^ and the electrolyte resistance (R_el_) are also included in the circuit. Note that in steady state and in the absence of Faradaic reactions at the electrode interfaces, *R_el_* may be neglected, and the two electrical double layer capacitances are replaced by their equivalent series capacitance (*C_gate_*).

We have developed a model to extrapolate the CNP of graphene by analyzing the experimental ω_*G*_ versus *V_G_* curves associated with the graphene G‐phonon Raman peak.

The relationship between the external bias, *V_G_* and the graphene's Fermi level, *E_F_* is:

(1) $$V_{G}=\frac{A_{g}e\;\left(n_{dop}-n\right)}{C_{gate}}+\frac{E_{F}}{e}$$

where *A_g_* is the graphene/water contact area, *n_dop_* is the initial carrier surface density, *n* is the carrier surface density concentration, and *e* is the elemental charge. The relationship between *E_F_* and *n* in graphene is:^[^ ^23^ ^]^

(2) $$\left|E_{F}\right|=\hbar v_{F}\;{\left(\pi \;\left|n\right|\right)}^{\frac{1}{2}}$$

where ℏ is the reduced Plank's constant and *v_F_* ≈ 1.1  ×  10^6^ m s^−1^ is the Fermi velocity. Eliminating *n* from Equations 1 and 2, we readily obtain the relationship between *E_F_* and the external bias *V_G_* as:

(3) $$E_{F}=\frac{\left[-\frac{C_{gate}}{A_{g}}+{\left({\left(\frac{C_{gate}}{A_{g}}\right)}^{2}+4\;\frac{e^{3}}{\pi \hbar^{2}v_{F}^{2}}\;\frac{C_{gate}}{A_{g}}\;\left|V_{G}-V_{CNP}\right|)\right)}^{\frac{1}{2}}\right]}{2\;\frac{e^{2}}{\pi \hbar^{2}v_{F}^{2}}\;sign\left(V_{G}-V_{CNP}\right)}\;$$

being $V_{CNP}=n_{dop}\;\cdot e\cdot \frac{A_{g}}{C_{gate}}$ the charge neutrality point (CNP) voltage, dependent on the initial doping state and the geometry of the device. Equation 3 correctly predict that the Fermi level coincides with the Dirac point at *V_G_* = *V_CNP_*  and graphene has zero charge carrier density and minimum electrical conductivity.^[^ ^9^ ^]^

Raman spectroscopy has emerged as a powerful technique for probing carrier density in graphene. The Raman spectrum of defect‐free graphene presents two main features: i) the G band corresponds to the in‐plane C─C stretching in sp^2^‐carbon materials; ii) the 2D band is associated with a second‐order double resonance process and gives information about the number of graphene layers.^[^ ^27^, ^28^ ^]^

Due to the strong electron‐phonon coupling characteristic of graphene, the G‐peak wavenumber in the Raman spectrum is highly sensitive to shifts in the Fermi level, relatively to the Dirac point. Particularly, when the Fermi energy falls in the [100–500] meV range with respect to the Dirac point, the following linear relationship holds:^[^ ^23^ ^]^

(4) $$\Delta \;\omega_{G}=\omega_{G}\;-\;\omega_{G}^{0}=\frac{\lambda_{\Gamma }}{2\pi }\;\left|E_{F}\right|$$

where $\omega_{G}^{0}$ = 1580 cm^−1^ is the G‐peak frequency for graphene at the charge neutrality point, and $\lambda_{\Gamma }$ is the electron‐phonon coupling constant, taken as 0.03.^[^ ^29^ ^]^

When the Raman spectrum of graphene is excited with a laser wavelength of 532 nm, the G and 2D band peaks depend on the value of the Fermi energy.^[^ ^30^ ^]^ Equations 3 and 4 show that the Δω_*G*_ shift is a function of either the applied voltage *V_G_* or the variation in the graphene charge neutrality point. The latter variation is expected during sensing experiments, when an affinity binding event induces an electrostatic change of the Au electrode surface potential and is detected via a capacitive coupling with the graphene transducing layer.

**Figure 2:**
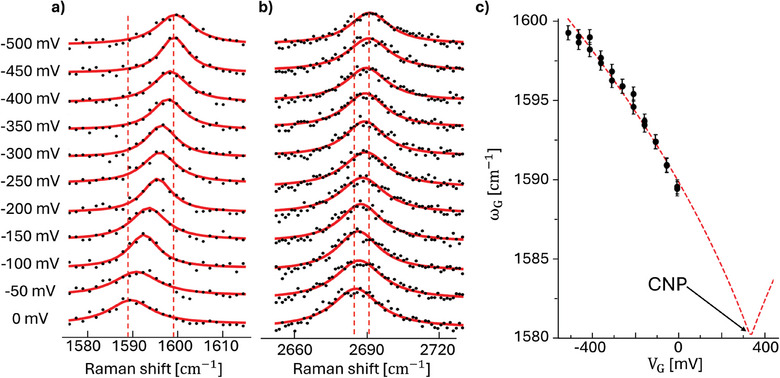
a) Raman spectra of G‐band of graphene as a function of the applied voltage *V_G_* as marked on the figure. The exciting laser wavelength is 532 nm. The solid lines are Lorentzian fits of the experimental bands. The Raman intensity (vertical) scale is the same for all spectra. The spectra are vertically offset for the sake of clarity. b) Same as panel (a) for the 2D‐band of graphene. c) G‐peak wavenumber position ω_*G*_ as a function of *V_G_* as extracted by fitting the Raman spectra of panel (a). The dashed line is the best fit obtained with Equations 3 and 4 (reduced χ^2^=1.26). The solid arrow indicates the extrapolated value of the charge neutrality point, CNP = (335 ± 11) mV.

**Figure** **2** illustrates the shift of the ω_*G*_ and ω_2*D*_ Raman peaks as a function of the applied voltage *V_G_*. At *V_G_* =  0 *V*, the G‐band is peaked at 1589 cm^−1^, which is blue‐shifted by ≈9 cm^−1^ with respect to the charge neutrality condition. The latter shift is a known sample dependent phenomenon in graphene, which is commonly ascribed to unintentional doping associated with structural defects in the 2D lattice.^[^ ^31^ ^]^ By applying a negative *V_G_* voltage, the Fermi energy of graphene decreases, and the G‐peak blueshifts further, reaching ≈1599 cm^−1^ at *V_G_* =   − 0.5 *V*. The measured relative shift is $\left(\frac{\Delta \omega_{G}}{|\Delta V_{G}|}\approx 20\;cm^{-1}\;V^{-1}\right)$, as shown in Figure 2a. The 2D peak exhibits a smaller value of the relative shift (*≈*14 cm^−1^ V^−1^) (see Figure 2b), also in agreement with previous reports.^[^ ^32^ ^]^ Accordingly, for sensing experiments we will concentrate on the G‐band.

Figure 2c shows that the measured ω_*G*_ versus *V_G_* is perfectly reproduced by our model based on Equations 3 and 4. The best fit gives an estimate of the gate capacitance, *C_gate_* = (1.72  ± 0.1) µF, comparable with typical values measured in water‐gated graphene devices,^[^ ^33^ ^]^ and a charge neutrality point CNP  = (335  ± 11) mV.

We select the immunoglobulin M (IgM) and its antibody (anti‐IgM) as a model binding pair for our investigations. The biofunctionalization protocol entails the physisorption of the capturing antibodies, directly onto the Au/SiO_2_/Si electrode (see Experimental Section). This simple and rapid method allows for the formation of a stable densely packed monolayer of (1.13 ± 0.04) × 10^12^ cm^−2^ capturing proteins, which fully covers the Au electrode surface. Furthermore, the physisorbed biolayer has been proved to have a shelf life of about 3 weeks, while retaining its biological activity.^[^ ^24^, ^34^ ^]^

**Figure 3:**
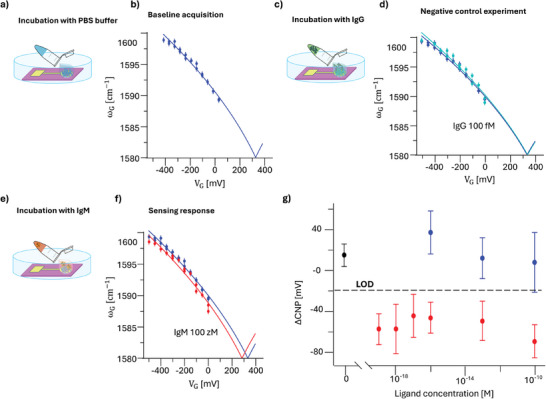
a) Schematic representation of the incubation of the anti‐IgM biofunctionalized Au electrode with PBS, 7.4, ionic strength 162 × 10^−3^ m, without affinity ligands; b)  ω_ g_ phonon wavenumber as a function of the applied voltage V_G_, as measured after the incubation step shown in panel a). The blue solid line is the best fit obtained using Equations 3 and 4. The extrapolated minimum phonon wavenumber corresponds to the graphene charge neutrality point (CNP); c) Same as (a) with IgG ligand concentration of 10^−13^ m; d) Same as panel (b) but measured after the incubation step shown in panel (c). The comparison with the data of panel (b) shows no significant shift of the CNP; e) Same as a) with IgM ligand concentration of 10^−19^ m; f) Same as panel (b) but measured after the incubation step shown in panel e). The comparison with the data of panel b) shows a significant shift of the CNP; g) CNP shift (Δ_CNP_) as a function of increasing concentrations of IgM (red dots) or IgG (blue dots). The data points are the average values over three replicates. Error bars are standard deviations. The black circle is the blank average sensor response. The dashed‐black line defines the limit of detection (LOD) (see Section S2, Supporting Information).

The sensing experiment flowchart is reported in **Figure** **3**a–f. The protocol begins incubating the Au/anti‐IgM slide for 10 min in plane phosphate‐buffered saline (PBS) solution (ionic strength = 163 × 10^−3^ m, 7.4). We chose to perform the incubation step outside the cell environment to prevent any nonspecific adsorption of ligands onto the graphene surface, which works as transducer (Figure 3a); the sensing electrode is then rinsed with deionized water and transferred back to the measurement cell, for the baseline acquisition.

The cell is connected to a voltage generator, which applies a DC voltage to the Au/anti‐IgM electrode within the range [−0.5, 0V], while maintaining the graphene electrode grounded. We selected a voltage range within the electrochemically inert region to prevent any faradaic current which might involve electron transfer reactions at the electrode surface, actually impacting protein layer stability.^[^ ^35^, ^36^, ^37^ ^]^ The Raman spectrum is recorded always on the same graphene electrode 5 µm‐wide spot, thereby minimizing possible variability due to inhomogeneities of the graphene. This is carried out by controlling the sample position under the Raman microscope during the sensing experiments, as described in Section S2 and Figure S2 (Supporting Information). The wavenumber position ω_*G*_ of the Raman G‐peak is extracted by Lorentzian fit of the peak, as a function of *V_G_*; then, Equations 3 and 4 are used to fit the dispersion of ω_*G*_ versus *V_G_* and the CNP is extracted as a fit‐parameter. The typical sensor response is reported in Figure 3b; for this specific sample we found a *CNP*  =  (335  ± 11) mV. The uncertainty on the CNP measurement represents an estimate of the associated standard deviation as provided by the fit algorithm. The reliability of CNP data for an Au/anti‐IgM sample has been proven by exposing the biofunctionalized electrode to ambient atmosphere for 5 h and subjected to several incubation/washing steps (See Figure S3, Supporting Information), as detailed in Section S3 (Supporting Information). These control experiments demonstrate that no significant change occurs in the CNP values, providing strong evidence that excludes the influence of any spurious effect on the CNP readings. The relevant data have been used to compute the sensor metrics, i.e., limit of detection (LOD) and limit of identification (LOI).

To test the sensor selectivity, our protocol includes negative control experiments in which the Au/anti‐IgM electrode is incubated in PBS solution containing increasingly high concentrations of immunoglobulin G (IgG) up to 10^−10^ m, corresponding to 10^7^ molecules in 0.1 mL (Figure 3c). The rationale behind this choice lies in the fact that IgG does not selectively binds to anti‐IgM although the two molecules share overlapping epitopes and similar molecular structures. The sensor response (light blue line) collected after the incubation of the sensing electrode with the interferent IgG is given in Figure 3d. Indeed, the resulting CNP (dark blue line) remains almost constant (340 ± 13) mV, within one standard deviation, when exposed to IgG.

Afterward, the same sensing electrode is transferred in the incubation well and exposed to the affinity antigen IgM solution 1 × 10^−19^ m (Figure 3e). The response is represented with a red solid line in Figure 3f, showing a significant shift of the CNP to the value (286 ±11 mV). The shifts in the charge neutrality point (ΔCNP) as a function of increasing concentrations of IgM (sensing assays) or non‐binding ligands (negative control experiments) are reported in Figure 3g. Even at these higher concentrations, no significative change in the CNP is found, with respect to the baseline. According to IUPAC definition, the LOD is defined as the average noise level from blank experiments (µ_n_ = 14 mV) plus three times its standard deviation (3σ = 11 mV), as detailed in Section S3 (Supporting Information). The dashed black line represents the LOD evaluated from the blank experiments. Remarkably, exposing the biolayer to as few as (6 ± 2) IgM molecules in 0.1 mL triggers a significative shift of ∆CNP = (−55 ± 15) mV, which falls below the LOD, at a confidence level of 99%. We found a limit of identification, LOI (6σ) of 66 mV.

Specifically, the relative shift, $\frac{\Delta \mathrm{CNP}}{\mathrm{CNP}}\%=\left(\frac{\mathrm{CN}P_{\mathrm{sensing}}-\mathrm{CN}P_{\mathrm{baseline}}}{\mathrm{CN}P_{\mathrm{sensing}}}\right)\%$, of ≈16% in the charge neutrality point, indicates a shift of the Fermi level toward the Dirac point of graphene; meaning that, a decrease in the p‐type doping level of the graphene occurs. Notably, increasing the ligand concentration does not further affect the electronic properties of the graphene electrode, thus resulting in an almost constant CNP shift of Δ*CNP*  = (− 55 ± 20)  mV, over concentrations 9 orders of magnitude larger.

**Figure 4:**
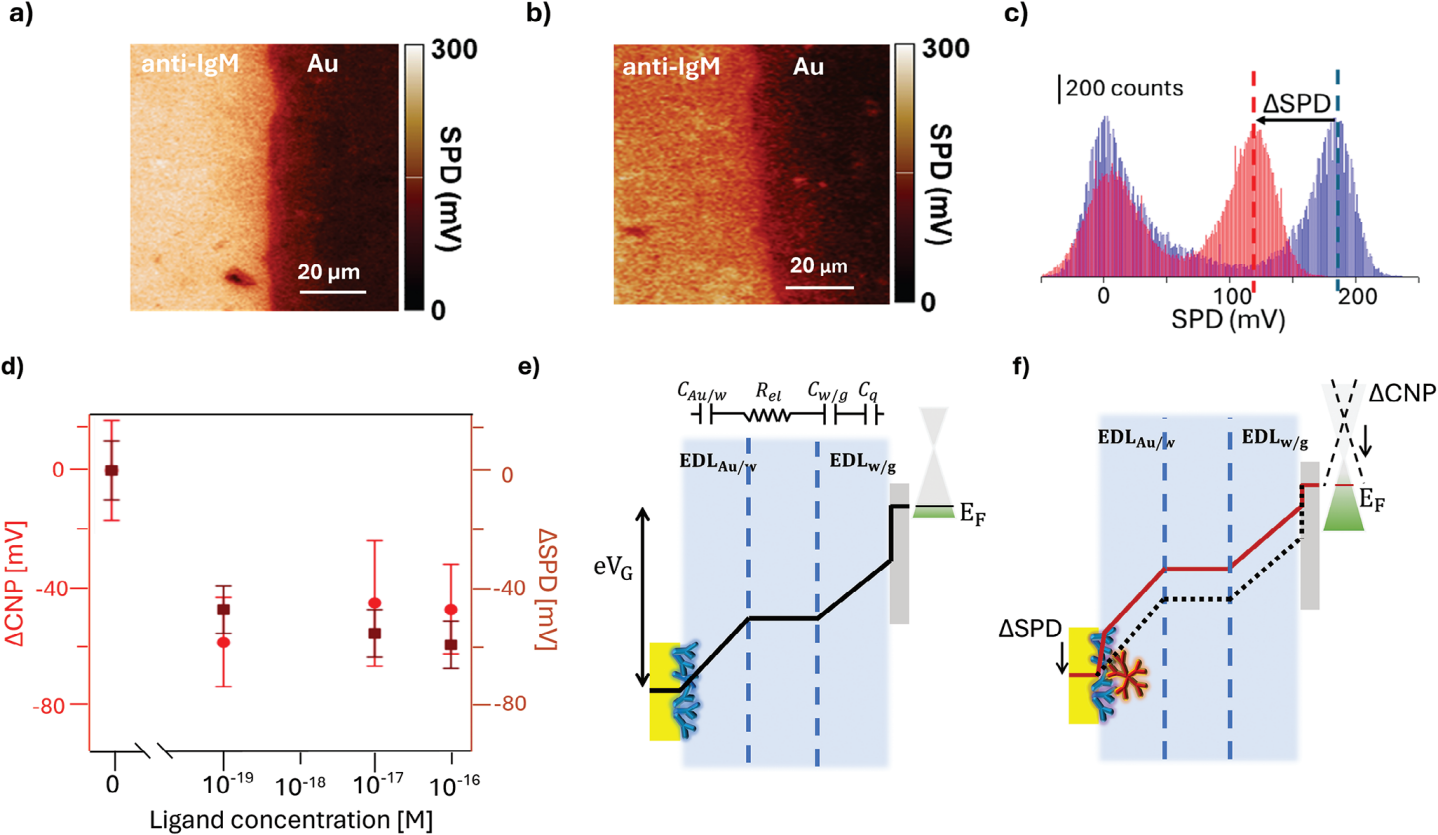
a) KPFM image of a pristine Au/anti‐IgM layer at the interface with the bare gold substrate. b) KPFM image of the sample area inspected in panel a) after the incubation with a standard solution of IgM 10^−19^ M. c) Comparison of the SPD histograms of images (a) and (b), showing a decrease of (− 66  ± 11) mV in the SPD after the occurrence of single‐molecule affinity bindings. The histogram of the pristine sample is blue while that after sensing experiment is red. d) Comparison between the ∆SPD and ∆CNP for Au/anti‐IgM electrode subjected to the same sensing protocol. The data points are the average values over two replicate experiments. Error bars are standard deviation. e,f) Schematic potential energy diagram across the device structure, along with the equivalent circuit. e) Before the selective binding of an antigen, the applied potential is distributed as voltage drop across the EDLs at the gold/water and water/graphene interfaces, and as a shift of the Fermi energy in graphene. f) After the affinity binding with IgM ligands the voltage drop is redistributed due to a change in the surface potential of the biolayer. This results in shifted Fermi level, at the same applied voltage V_G_. The characteristic band structure of graphene (Dirac cone) is sketched on the right side of panels (e) and (f), with the green part showing occupied states.

To provide essential pieces of information, the Au/anti‐IgM electrode has been inspected by kelvin probe force microscopy. KPFM is a widely used technique, which analyzes with nanometric spatial resolution, changes in work function for metal or surface potential for non‐metals by measuring contact potential difference between the sample surface and a conducting cantilever tip. Here, we use KPFM to directly assess the surface potential shift of the biofunctionalized Au/anti‐IgM sensing electrode upon single‐molecule binding events and further correlate it with the already mentioned shift of the graphene charge neutrality point. The KPFM analysis is performed across the interface between the biofunctionalized and the pristine areas of a patterned Au/anti‐IgM electrode, following the procedure described in the literature^[^ ^21^, ^34^ ^]^ and detailed in the Experimental Section. The sensing experiments involve the following steps: i) incubation with phosphate buffer solution (PBS, 7.4, ionic strength 162 × 10^−3^ m, serving as baseline; ii) incubation for 10 min of the same Au/anti‐IgM sample with increasing concentration of IgM in PBS (10^−19^, 10^−17^, and 10^−16^ m); iii) KPFM images recording at the same scanning area, at each step of the sensing protocol. The Surface Potential Difference (SPD) across the interface between the biolayer and the gold reference area is used as the analytical parameter.^[^ ^21^, ^34^ ^]^ **Figure** **4**a,b features the representative 90 × 90 µm^2^ KPFM surface potential landscapes before and after the occurrence of the recognition process in the single‐molecule regime (6 ± 2 IgMs in 0.1 mL), respectively. In **Figure** **4**c, the corresponding SPD distribution hystograms are given. The data show that the formation of a few immuno‐complexes induces a significant change in the contrast of the entire KPFM image with respect to the pristine Au/Anti‐IgM image. A decrease of (− 66  ± 11) mV in the SPD has been measured for this sample. We hypothesized that the conformational change caused by the biochemical binding alters the electrostatic properties of the capturing antibody, leading to a shift in the overall dipole moment or causing it to become misaligned. Furthermore, we propose that this localized alteration influences neighboring antibodies due to the close packing of recognition elements on the surface. The interaction occurs through a cooperative electrostatic network, where changes in one antibody propagate to others nearby.^[^ ^21^, ^34^ ^]^ Figure 4d presents a comparative analysis of the measured shift in surface potential difference (∆SPD), along with shifts in charge neutrality point (∆_CNP_) for Au/anti‐IgM electrodes subjected to the same sensing protocol. The data are averaged from at least two replicates, each using two different Au/anti‐IgM substrates, while the error bars are given as one standard deviation. An average decrease Δ*SPD*  = (− 54 ± 10) mV was found across a three‐order‐of‐magnitude concentration range, in the inspected biosystem. This is in quantitative agreement with the shift in graphene charge neutrality point voltage measured in the graphene‐based sensor.

The shift in surface potential,^[^ ^34^ ^]^ leads to a variation in the doping level of the graphene electrode. In other words, the changes in surface potential induced by the affinity binding of a few antigens are effectively transduced and amplified by the graphene, even at single‐molecule detection level. Figure 4e schematically shows the potential energy across our device, before and after sensing, that is compared to the ∆SPD shifts (Figure 4f).The work function of a metal could be expressed as the sum of a bulk contribution and a surface electrostatic potential term, due to a layer of surface dipoles with negative charges oriented toward the vacuum side.^[^ ^38^ ^]^ The physical absorption of a densely packed capturing film affects the dipole surface potential term. When a negative bias is applied to the biofunctionalized Au electrode with respect to graphene, (see inset of Figure 4e) the potential drop is shared among the two charge double layers, and the quantum capacitance of graphene. Our findings show that the sensing platform works as an “OFF/ON” detector. In the absence of target molecules, the sensor remains in an “OFF” state, characterized by an initial p‐doping level of the graphene layer. Upon exposure to the target analyte, the sensor turns to an “ON” state, showing a significant change in the graphene Fermi level.

This is consistent with our previous results using transistor‐based sensing technologies, named Single Molecule with large Transistor (SiMoT) platform. SiMoT has been proven capable of reliable detection of single molecules in 100 µL of assay solution for both genomic and protein biomarkers, (e.g., miR‐182, IgG, IgM, HIV‐1, P24, and CRP) showing a LOD as low as 10^−20^ M, even in real biofluids.^[^ ^20^, ^35^, ^39^, ^40^, ^41^, ^42^ ^]^ In the SiMoT platform, the millimetric gate electrode surface is biofunctionalized with an high density of capturing molecules (10^12^ receptors∙cm^−2^), enabling detection within 10 min timeframe. We proposed a domino‐like mechanism whereby not only the single antibody involved in the recognition process, but also trillions of capturing sites on the sensing electrode contribute to cooperative signal amplification. This is further supported by a model based on the Einstein diffusion theory, demonstrating that a single‐molecule out of a few diffusing in a 0.1 mL volume has a high probability to hit a large capturing sensing electrode functionalized with trillions of recognition elements within 10 min and bind to a capturing protein.^[^ ^43^ ^]^

The same amplification effect is assumed in the graphene‐based sensor, meaning that the affinity binding events induce an extended change in the surface potential of the biofuctionalized electrode. Due to capacitive coupling through the electrolyte, the variations in surface potential are effectively transduced at the graphene electrode, resulting in a shift of the graphene Fermi level. Consequently, this shifts the graphene phonon frequencies, which are measured by Raman spectroscopy. Additionally, the integration of an electrolyte‐soaked paper strip coupling the biofunctionalized Au and the graphene electrode holds great promise for applications in low‐cost lateral flow immunoassays.

## 3 Conclusion

Our novel graphene‐based biosensor demonstrates a proof‐of‐concept label‐free detection of IgM with a sensitivity reaching a remarkably low concentration of 10^−19^ m. By utilizing a unique sensor architecture that combines a bio‐functionalized gold interface and a bare graphene electrode through a water‐soaked paper strip, this design achieves high sensitivity and flexibility in biomarker detection. The ability to directly measure changes in graphene phonon frequencies via Raman spectroscopy, as influenced by surface potential variations at the biofunctionalized interface, underscores the sensor innovative transduction mechanism to detect biomarkers by rapid optical probing. Our opto‐electronic sensor architecture relies on a two‐terminal electric scheme, which is considerably simpler than the transistor‐like one, and minimizes potential sources of noise, thereby potentially enhancing the sensor reliability. Moreover, the use of an electrolyte‐soaked paper strip for the coupling between the biofunctionalized Au and the graphene electrodes provides an effective means of maintaining a stable liquid environment, aligning with the growing interest in developing self‐contained, pump‐free microfluidic platforms driven by capillary forces. Such a sensor design choice, coupled with the unique optoelectronic properties of graphene and the powerful probing capabilities of Raman spectroscopy, has enabled us to achieve single molecule regime sensitivity in label‐free biomarker detection. In perspective, the increasing availability of portable Raman spectrometers opens to the development of a portable graphene‐based platform.

The performance of our device not only proves upon the capabilities of existing graphene‐based sensors through its inherent simplicity opto‐electronic integration but paves the way for future advancements in high‐performance diagnostic tools, establishing a new standard in the field of portable point‐of‐care diagnostics.

## 4 Experimental Section

### Materials

HPLC grade water, 30% (w/w) VLSI‐grade hydrogen peroxide, and 96% VLSI‐grade sulfuric acid, all obtained from Avantor, were used without any additional purification processes. Anti‐human immunoglobulin M (anti‐IgM), human IgM (≈950 kDa), and IgG (≈150 kDa) were also purchased from Sigma–Aldrich–Merck. All the immunoglobulins used in this study were polyclonal antibodies. Phosphate‐buffered saline (PBS) solutions had a pH of 7.4 and an ionic strength of 162 mM.

### Chemical Vapor Deposition of Graphene and Transfer

Graphene was grown on a (25 µm) copper foil of (10 × 10 cm^2^) size by metal catalyzed CVD methodology in a quartz tube furnace. The quartz‐tube was evacuated to a vacuum better than (10^−3^) torr and heated to 990 °C under an H_2_ gas flow of (10 sccm, 0.05 torr). Then, a (5 sccm) flow CH_4_ was added to the gas feed for the growth phase. After 20 min, the furnace was moved from the growth‐zone to realize the rapid cooling of the graphene/copper foil. Single layer CVD‐graphene was then transferred to the Corning‐glass substrate using the thermal release tape procedure. The sized sheet of TR‐tape was applied on a piece of graphene/copper foil then pressed by the laminator. Copper was removed in an ammonium persulfate solution (20 g L^−1^) and the floating sheet of graphene/TR‐tape was rinsed in deionized water and air dried. The graphene was directly transferred onto the substrate by hot‐pressing of the graphene/TR‐tape (at ≈100 °C) thus releasing the tape.

### Sample Preparation Protocol

Gold electrodes were prepared by electron beam evaporation of (50 nm) Au layer, using a (5 nm) Ti film as adhesion‐promoter on Si/SiO_2_ wafers, through a shadow mask. The resulting Au electrodes comprise a (20 mm^2^) circular pad, serving as the sensing area, a squared pad used for electrical connections, with the two pads linked by a (0.25 µm)‐thick channel. The substrates were cleaned with a piranha solution (H_2_SO_4_:H_2_O_2_ = 3:1), rinsed in abundant HPLC water, and dried with nitrogen. The gold gate electrodes for the G‐FET devices undergo an additional 10‐min cleaning step in a UV‐ozone chamber. However, this cleaning procedure was not carried out for the patterned substrates that were characterized by KPFM, to maintain the chemical inertness of the gold, which served as internal reference. This also ensured a well‐defined interface between the gold and the surrounding biolayer. The biofunctionalization was performed by dipping the samples into a PBS solution of anti‐IgM (0.1 mg mL^−1^) for 150 min, at room temperature. This protocol was demonstrated to reliably produce a stable densely packed layer of antibodies fully covering the electrode surface.^[^ ^24^ ^]^ The electrode was first rinsed with PBS solution to remove any unbounded molecules, and then HPLC water before being assembled into the G‐FET cell.

### Standard Solutions of Target Molecules

The standard solutions of IgM and IgG in PBS were prepared by serial dilution from mother solutions with a concentration of (5  ×  10^−5^)M. The stock solution was used for subsequent dilutions in a 100‐fold serial dilution protocol. The number of molecules is given by:

(5) $$\#\;\mathrm{molecules}\;=\;c\cdot N_{A}\cdot V$$

Being *c*, the concentration of analyte, *N_A_* the Avogadro Number and V, the solution volume.

Therefore, (6 ± 2) molecules are found in 0.1 mL of 1 × 10^−19^ m analyte solution.

### Raman Spectroscopy Measurements and Single Molecule Sensing

Raman spectra of graphene were measured by a NT‐MDT micro‐Raman setup, with a confocal 20X objective (NA = 0.25), in a backscattering configuration. The sample was illuminated by an unpolarized green laser (λ  =  532 nm), focused on a spot 5 µm in diameter; the laser power was kept below 3 mW and the exposure time was 100 s for each spectrum. Scattered light was dispersed by a grating monochromator with 4 cm^−1^ resolution and measured by a Peltier cooled CCD camera. Measurements were taken in ambient condition while room temperature is ≈25 ^○^ *C*.

The device output was the G‐peak central wavenumber of the Raman spectrum, measured as a function of the applied external bias. The bias was swept from 0 to −0.5 V, with a step size of 0.05 V. At each bias step, the Raman spectrum was recorded after maintaining the bias constant for 60 s. This allowed any transient currents in the device to relax before the measurement was taken.

After completing the full potential sweep, the charge neutrality point of the graphene was computed by fitting the data to the analytical model described by Equations 3 and 4. During each step of the sensing protocol, the Au/anti‐IgM electrode was removed from the measurement cell. It was incubated for 10 min in 0.1 mL of the analyte standard solution. After incubation, the sensing gate was rinsed with HPLC water to remove any unbound molecules and repositioned back into the measurement cell for the Raman analysis.

### Kelvin Probe Force Microscopy Analysis and Single Molecule Detection

The immobilization of the capturing antibodies on the gold sample was carried out by covering a portion of the substrate with a polymeric mask, allowing the physisorption of a densely packed IgM layer on the uncovered area of the sample.^[^ ^21^, ^34^ ^]^ The samples were investigated at room temperature, after drying by spin coating (3000 rpm, 60 s). The kelvin probe force microscopy analysis was performed using an NT‐MDT NTEGRA system (Moscow, Russia). The images were recorded in amplitude modulation two‐pass mode, with a platinum/iridium tip (TipsNano, mod. FMG01/Pt, apex size of 35 nm, resonance frequency f = 70 kHz), setting the tip‐sample distance at 250 nm. Morphology, phase and surface potential landscapes were recorded simultaneously over the same (90 × 90 µm^2^) scanning area at the interface between the pristine gold region and the biofunctionalized one. The analytical parameter was the surface potential difference (SPD) between the Au/anti‐IgM area and the pristine gold region, using the latter as internal reference. All images were processed using the Image Analysis software. The single‐molecule sensing experiments include the following steps: the Au/anti‐IgM was deep coated in the 0.1 mL PBS standard solution to be assayed for 10 min at room temperature, serving as baseline. Then the negative control experiment was performed, carried out in a 10^−13^ m solution of the non‐binding IgG. The electrode was washed thoroughly with HPLC water and biased against a gold counter electrode in HPLC water and cycled, afterward, by sweeping the potential in the [0–(−0.5)] V range, for 20 cycles. After drying, the sample was inspected by KPFM. The same biosystem was sequentially immersed in 0.1 mL of a PBS standard solution with increasing concentration of IgM for 10 min. After each incubation the electrode was washed, cycled, dried and assessed by KPFM.

### Statistical Analysis

The Raman spectroscopy data analysis was performed using a custom Python script. Raman spectra of graphene were subject to a second order polynomial background subtraction in the G‐peak (1550–1680 cm^−1^) and 2D‐peak (2600–2830 cm^−1^) spectral region. The G‐peak wavenumbers were estimated by fitting a Lorentzian function, and an error bar of 0.4 cm^−1^ was associated to the extracted value (one standard deviation over 15 replicate measurements). Each set of data, including the ω_*G*_ versus V_G_ measurement was analyzed by a fit using Equations 3 and 4. All fit returned a root mean square error always lower than 1 cm^−1^, suggesting a good reproduction of the experimental data. The ΔCNP values reported in Figure 3g summarize the outcomes from three sensing experiments and one negative control experiment. The measured ΔCNP values for each sensing experiment, and for the negative control experiment are reported in **Tables** **1** and **2** respectively.

**Table 1:** ΔCNP values measured in each of the three sensing experiments, after exposure of the biofunctionalized Au electrode to increasing concentration of IgM solution.

ΔCNP	[IgM] = 10^−19^ M	[IgM] = 10^−18^ M	[IgM] = 10^−17^ M	[IgM] = 10^−16^ M	[IgM] = 10^−13^ M	[IgM] = 10^−10^ M
Sensing exp.1	(−49 ± 16) mV	/	/	(−59 ± 16) mV	(−48 ± 16) mV	(−70 ± 16) mV
Sensing exp.2	(−66 ± 25) mV	(−58 ± 24) mV	(−62 ± 24) mV	(−50 ± 25) mV	/	/
Sensing exp.3	/	/	(−28 ± 35) mV	(−33 ± 34) mV	(−52 ± 35) mV	(−93 ± 31) mV

**Table 2:** ΔCNP values measured in the negative control experiment, after exposure of the biofunctionalized Au electrode to increasing concentration of unbinding IgG solution.

ΔCNP	[IgG] = 10^−16^ M	[IgG] = 10^−13^ M	[IgG] = 10^−10^ M
Neg. cont. exp	(36 ± 21) mV	(11 ± 20) mV	(7 ± 19) mV

The KPFM images were processed using Image Analysis. From each image, the SPs of the gold area, SP_Au_, and the biolayer one, SP_Bio_, are evaluated as the mean of the SP distributions. In the raw data, because of surface contamination by airborne adventitious carbon,^[^ ^34^ ^]^ the gold surface potential may vary between measurements. To ensure consistency and enable standardized analysis across datasets, the mean value of SP_Au_ was fixed at 0 mV, aligning all measurements to a common reference. SP_Bio_ was estimated through a Gaussian fit of the SP histograms using Origin software, with its corresponding standard deviation.

The resulting SPD was computed as:

(6) $$\mathrm{SPD}\;=\left(SP_{\mathrm{Bio}}-SP_{\mathrm{Au}}\right)$$

The ΔSPD values (variations upon exposure of the functionalized Au electrode to analyte solutions). **Table** **3** shows the ΔSPD values measured in each individual experiments.

**Table 3:** ΔSPD values measured in each of the two sensing experiments, after exposure of the biofunctionalized Au to increasing concentration of IgM solution.

ΔSPD	[IgM] = 10^−19^ M	[IgM] = 10^−17^ M	[IgM] = 10^−16^ M
Sensing exp. 1	(−43 ± 12) mV	(−59 ± 13) mV	(−60 ± 13) mV
Sensing exp. 2	(−50 ± 10) mV	(−50 ± 10) mV	(−57 ± 10) mV

## Conflict of Interest

The authors declare no conflict of interest.

## Supporting information



Supporting Information

## Data Availability

The data that support the findings of this study are available from the corresponding author upon reasonable request.
