# Supplementary material for: Graphene‐Based Opto‐Electronic Platform for Ultra‐Sensitive Biomarker Detection at Zeptomolar Concentrations
Source: Small Methods. 2025 Jan 21;9(8):2402026. doi: 10.1002/smtd.202402026 (PMC12391637; doi:10.1002/smtd.202402026)
Supplement: Supplementary file 1 — Supporting Information [file SMTD-9-2402026-s001.docx]

SUPPORTING INFORMATION

**Graphene-Based Opto-Electronic Platform for Ultra-Sensitive Biomarker Detection at Zeptomolar Concentration**

Matteo Piscitelli, Cinzia Di Franco*, Giuseppe Valerio Bianco, Giovanni Bruno, Eleonora Macchia, Luisa Torsi, and Gaetano Scamarcio*

**S1. Graphene-based sensor layout**

The graphene sensor is a two-terminal device consisting of a Si/SiO2/Au electrode, and a single-layer graphene electrode, capacitively coupled via a soaked paper strip. The picture of the device is given in Figure S1. The Au electrode consists of 5/50 nm of Ti/Au evaporated on a Si/SiO2 substrate using e-beam evaporation; the gold pattern is defined using a shadow mask, featuring a 20 mm^2^ circular pad and a square pad connected by a narrow channel. The circular pad serves as sensing area and is bio-functionalized with a monolayer of physisorbed anti-Immunoglobulin M (anti-IgM). The gate electrode is placed upside-down, with the functionalized pad facing the soaked paper strip, while the square pad contacts the Au/Ti/Kapton electrode, providing electrical connection to the voltage generator. The paper strip is placed on a Si/SiO_2_ substrate, with one edge submerged in a water reservoir (not shown), creating a water channel between the sensing Au electrode and the graphene electrode. The graphene electrode is made of CVD-grown single-layer graphene, transferred onto a transparent glass slab. It is positioned on the Si/SiO_2_ substrate, ensuring that the water remains confined within the edge of the substrate, wetting a controlled area of the graphene (~20 mm²). The opposite edge of the graphene is connected to a voltage generator via an Au/Ti/Kapton electrode. The entire device is secured to a plastic holder, using clamps. During experiments, the base is fixed under the Raman microscope objective to ensure high reproducibility when measuring the Raman spectrum of the graphene. This design enables ex-situ incubation of the electrode gate, as it can be easily detached and reassembled.


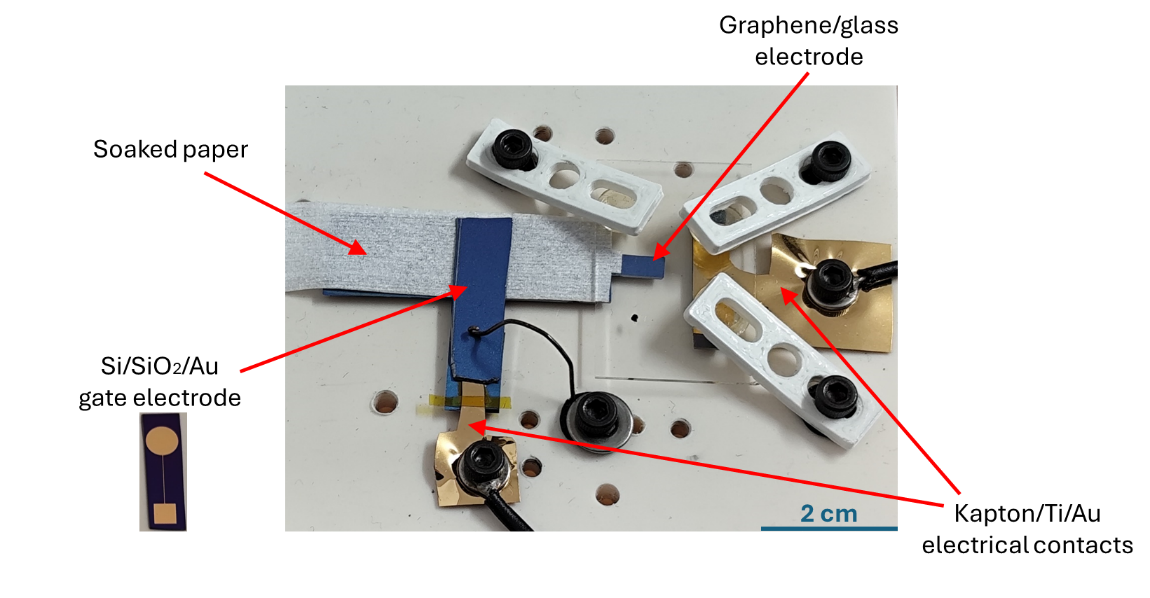


***Figure S1.*** *Picture of the device. It includes a Si/SiO_2_/Au electrode, a soaked paper strip, a CVD single-layer graphene electrode deposited on a glass slab; electrical connection to the gold and graphene are provided by kapton/Ti/Au soft electrodes, which are connected to the voltage generator. The entire setup is secured with clamps and mounted on a plastic holder.*

**S2. Raman spectroscopy characterization of graphene layer**

The Raman spectrum of the graphene layer on the glass substrate, shown in **Figure S2a,** displays the characteristic G-peak (⁓1590 cm⁻¹) and 2D-peak (⁓2700 cm⁻¹). The 2D-peak intensity is twice the G-peak one, confirming that the sample consists of single-layer graphene. The optical microscope image in **Figure S2b** reveals that the graphene surface is relatively smooth and uniform, without macroscopic cracks or defects. Local variations in the Raman spectrum of graphene were investigated, using Raman imaging over 20x20 µm² areas. Specifically, the wavenumber position of the G-peak ($\omega_{G}$) and the intensity ratio $I_{2D}/I_{G}$ were recorded, as shown in **Figure S2c** and **Figure S2d**, respectively. **Figure S2c** shows that $\omega_{G}$varies by about 4 cm^-1^ across the scanned area, indicating potential inhomogeneities that could affect the device reproducibility. To avoid this, the sample position under the Raman microscope is controlled, ensuring that the spectra were consistently acquired always from the same 5 µm spot on the graphene surface throughout the sensing experiment. On the other hand, **Figure S2d** shows that the intensity ratio $I_{2D}/I_{G}$is > 1, further confirming that graphene is single layered across the whole scanned area.”


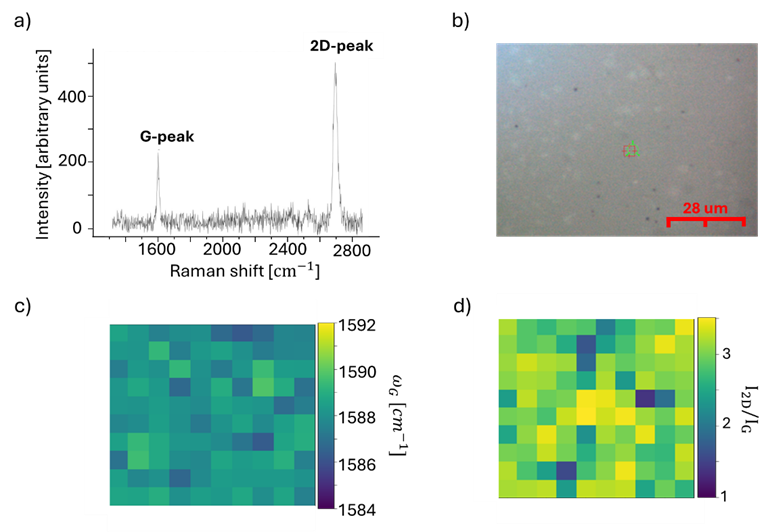


***Figure S2.*** *Raman spectroscopy analysis of the CVD graphene.* ***a)*** *Raman spectrum of the CVD graphene transferred on glass. The spectrum features the G-peak (⁓1590 cm-1) and the 2D-peak (⁓2700 cm^-1^).* ***b)*** *Optical image of the graphene on glass.* ***c)*** *Imaging of the wavenumber position* $\omega_{G}$*of the graphene G-peak.* ***d)*** *Imaging of the intensity ratio* $I_{2D}/I_{G}$*. Both images are recorded over a 20x20 µm^2^ area.*

**S3. *Blank experiments***

The stability of the graphene-based biosensor was assessed through a series of blank experiments. The analysis underwent four blank experiments using phosphate buffer solution (PBS, ionic strength 162 mM, pH 7.4) over a period of 5 hours. Each experiment involved exposing the Au/anti-IgM electrode in PBS for 10 minutes in the incubation well, followed by thorough rinsing in deionized water. After each washing the Au/anti-IgM is placed in the measurement cell. The cell is connected to a voltage generator, which applies a voltage to the Au/anti-IgM gate in the range [0 to -0.5] V, while keeping the graphene electrode grounded. The Raman spectrum is recorded, and the graphene charge neutrality point was computed using the developed method. The results show that the CNP values remained nearly constant throughout the control experiment, with an average ∆CNP of (14 ± 11) mV. The blank experiments were customarily used to compute sensor metrics. According to IUPAC definition, the limit of detection (LOD) is defined as the average noise level from blank experiments (µ_n_ = 14 mV) plus three times the standard deviation (3σ = 11 mV), while the limit of identification (LOI) is the average noise level plus six times the standard deviation (6σ = 66 mV).


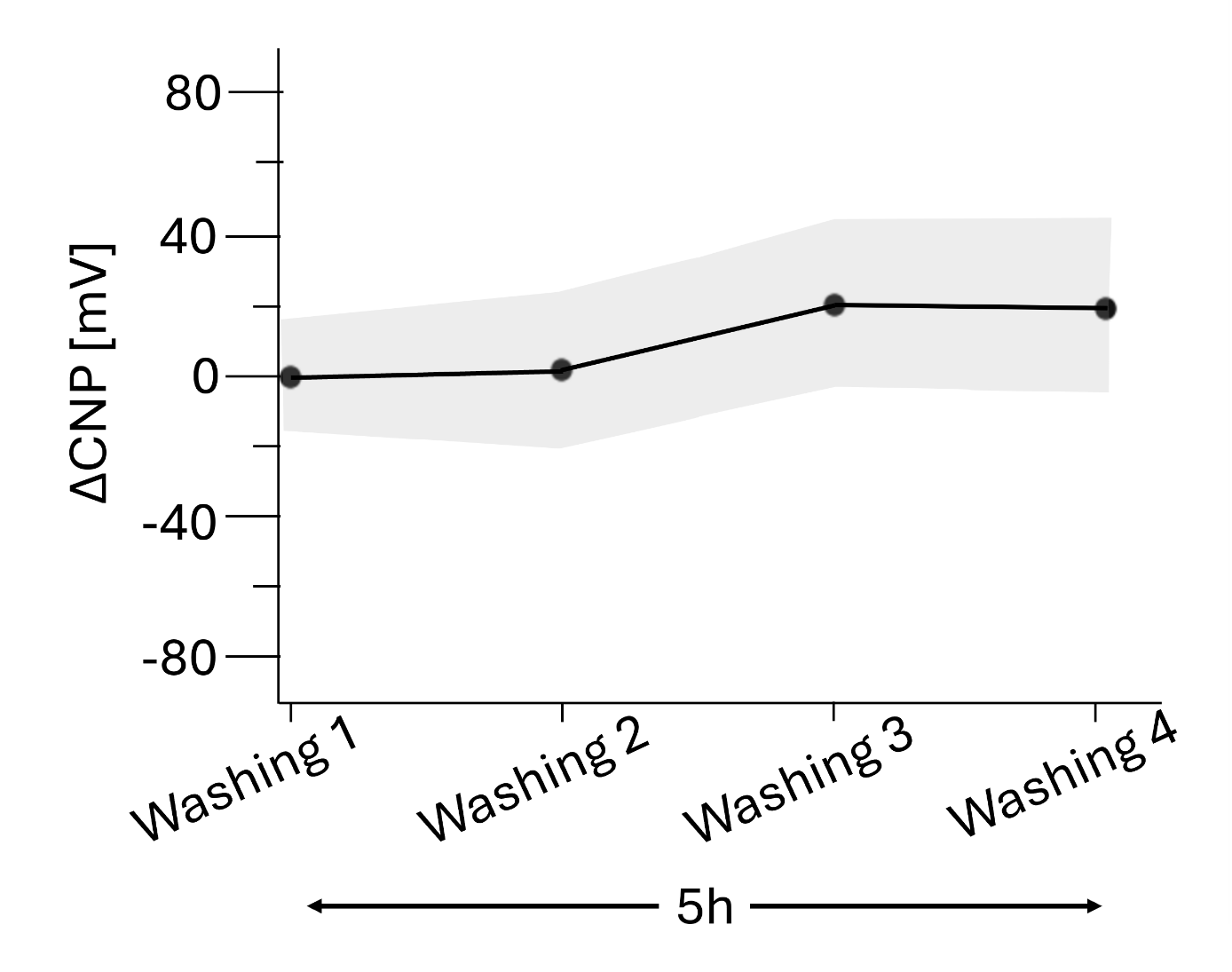


***Figure S3.*** *Shift in the graphene charge neutrality point as a function of repeated incubation/washing steps in plain PBS, within a 5-hour period. The colored shading represents one standard deviation*.
